# Sex-based clinical and immunological differences in COVID-19

**DOI:** 10.1186/s12879-021-06313-2

**Published:** 2021-07-05

**Authors:** Bin Huang, Yun Cai, Ning Li, Kening Li, Zhihua Wang, Lu Li, Lingxiang Wu, Mengyan Zhu, Jie Li, Ziyu Wang, Min Wu, Wanlin Li, Wei Wu, Lishen Zhang, Xinyi Xia, Shukui Wang, Hongshan Chen, Qianghu Wang

**Affiliations:** 1 Center for Global Health, School of Public Health, Nanjing Medical University, Nanjing, 211166 Jiangsu China; 2 Department of Bioinformatics, Nanjing Medical University, Nanjing, 211166 Jiangsu China; 3 Key Laboratory of Cardiovascular & Cerebrovascular Medicine, School of Pharmacy, Nanjing Medical University, Nanjing, China; 4 Department of Laboratory Medicine & Blood Transfusion, the 907th Hospital, Nanping, 350702 Fujian China; 5 Joint Expert Group for COVID-19, Department of Laboratory Medicine & Blood Transfusion, Wuhan Huoshenshan Hospital, Wuhan, 430100 China; 6 COVID-19 Research Center, Institute of Laboratory Medicine, Jinling Hospital, Nanjing University School of Medicine, Nanjing Clinical College of Southern Medical University, Nanjing, 210002 Jiangsu China; 7 Department of Laboratory Medicine, Nanjing First Hospital, Nanjing Medical University, Nanjing, 210006 Jiangsu China; 8 Department of Cardiothoracic Surgery, the Second Affiliated Hospital of Nanjing Medical University, Nanjing, China; 9 Key Laboratory of Targeted Intervention of Cardiovascular Disease, Collaborative Innovation Center for Cardiovascular Disease Translational Medicine, Nanjing Medical University, Nanjing, China; 10 State Key Laboratory of Reproductive Medicine, Nanjing Medical University, Nanjing, 211166 China

**Keywords:** COVID-19, Sex, Immunology, Prognosis, SARS-CoV-2

## Abstract

**Background:**

Males and females differ in their immunological responses to foreign pathogens. However, most of the current COVID-19 clinical practices and trials do not take the sex factor into consideration.

**Methods:**

We performed a sex-based comparative analysis for the clinical outcomes, peripheral immune cells, and severe acute respiratory syndrome coronavirus (SARS-CoV-2) specific antibody levels of 1558 males and 1499 females COVID-19 patients from a single center. The lymphocyte subgroups were measured by Flow cytometry. The total antibody, Spike protein (S)-, receptor binding domain (RBD)-, and nucleoprotein (N)- specific IgM and IgG levels were measured by chemiluminescence.

**Results:**

We found that male patients had approximately two-fold rates of ICU admission (4.7% vs. 2.7% in males and females, respectively, *P* = 0.005) and mortality (3% vs. 1.4%, in males and females, respectively, *P* = 0.004) than female patients. Survival analysis revealed that the male sex is an independent risk factor for death from COVID-19 (adjusted hazard ratio [HR] = 2.22, 95% confidence interval [CI]: 1.3–3.6, *P* = 0.003). The level of inflammatory cytokines in peripheral blood was higher in males during hospitalization. The renal (102/1588 [6.5%] vs. 63/1499 [4.2%], in males and females, respectively, *P* = 0.002) and hepatic abnormality (650/1588 [40.9%] vs. 475/1499 [31.7%], *P* = 0.003) were more common in male patients than in female patients. By analyzing dynamic changes of lymphocyte subsets after symptom onset, we found that the percentage of CD19+ B cells and CD4+ T cells was generally higher in female patients during the disease course of COVID-19. Notably, the protective RBD-specific IgG against SARS-CoV-2 sharply increased and reached a peak in the fourth week after symptom onset in female patients, while gradually increased and reached a peak in the seventh week after symptom onset in male patients.

**Conclusions:**

Males had an unfavorable prognosis, higher inflammation, a lower percentage of lymphocytes, and indolent antibody responses during SARS-CoV-2 infection and recovery. Early medical intervention and close monitoring are important, especially for male COVID-19 patients.

**Supplementary Information:**

The online version contains supplementary material available at 10.1186/s12879-021-06313-2.

## Background

The outbreak of coronavirus disease 2019 (COVID-19) caused by severe acute respiratory syndrome coronavirus 2 (SARS-CoV-2) infection is a pandemic, spreading to more than 210 countries and regions [1, 2]. As of April 7, 2021, a total of 132,046,206 confirmed cases were reported, of which 2,867,242 patients died (WHO situation report), extremely challenging the public health and medical service around the globe. Investigating the risk factors of susceptibility and prognosis for COVID-19 was necessary to help disease prevention and precise therapy.

According to previous studies, age is a risk factor for death from COVID-19 patients [3]. In a report of 1099 patients with COVID-19 from 552 hospitals in 30 provinces in China, patients with severe disease were older than those with the non-severe disease by a median of 7 years [4]. SARS-CoV-2 and SARS-CoV have more than 85% identical nucleic acid sequences [5]. Therefore, the epidemiological risk factors may be similar between SARS-CoV and SARS -CoV-2. In addition to age, epidemiological studies showed that the incidence and mortality of SARS-CoV infection were sex-dependent [6]. Males were more susceptible and experienced more severe disease after SARS-CoV infection [7]. A recent case series study reported that 75% of patients who died of COVID-19 were male [8]. Some researchers proposed that clinical trials for COVID-19 should include sex as a variable because of the biological difference between males and females [9]. Experiments in mice indicated that ovariectomy or treating female mice with an estrogen receptor antagonist increased mortality after SARS-CoV infection [10], suggesting the hormonal effect plays an important role in the immune response against infection. Takahashi T et al. reported that male COVID-19 patients had higher innate cytokines, lower T cell response, and higher mortality compared with female patients [11]. Although some studies reported differences in clinical outcomes between male and female COVID-19 patients, due to insufficient test data samples, a comprehensive analysis of the underlying cellular and molecular mechanisms was not conducted. In this study, by describing the clinical and laboratory characteristics of 3057 COVID-19 patients from a single center, we performed a sex-based comparative analysis for the clinical, cellular, and molecular differences in COVID-19. Our results provide important information for the epidemiology and precise therapy for this emergent pandemic.

## Methods

### Patients

We analyzed the laboratory test results of 3057 COVID-19 patients, including 1455 mild or moderate, 1417 severe, 150 critical, and 35 unclassified cases, admitted to Wuhan Huoshenshan Hospital from February 4 to March 30, 2020. A total of 3051/3057 (99.8%) patients were older than 18 years old. The severity degree of each patient was determined according to the clinical classification criterion in Diagnosis and Treatment Protocol for Novel Coronavirus Pneumonia released by the National Health Commission (trial version 7; http://en.nhc.gov.cn/2020-03/29/c_78469.htm). Patients who met any of the following criteria were diagnosed as severe cases: (1) shortness of breath defined by respiration rate ≥ 30 breaths/min, (2) oxygen saturation ≤ 93 at rest, and (3) alveolar oxygen partial pressure/fraction of inspiration O2 (PaO2/FiO2) ≤ 300 mmHg (1 mmHg = 0.133 kPa). Patients whose pulmonary imaging showed significant progression of lesions > 50% within 24–48 h were also treated as severe cases. Patients who met any of the following conditions were diagnosed as critical cases: (1) respiratory failure requiring mechanical ventilation, (2) shock, and (3) organ failure needing intensive care unit (ICU) monitoring and treatment. Also, the severity degree of each patient in this study was defined as the most serious disease state during hospitalization. We obtained the clinical characteristics and laboratory findings of all patients from the electronic medical records of the hospital. This study was approved by the Medical Ethical Committee of Wuhan Huoshenshan Hospital. Written informed consent was obtained from each patient. The summary of necessary information (Supplementary Data Sheet 1), biochemical indicators (Supplementary Data Sheet 2), immune phenotype (Supplementary Data Sheet 3) and antibody level (Supplementary Data Sheet 4) were provided. High-dose steroids and tocilizumab were not used in this cohort, while the information about the use of low-dose steroids in patients (Supplementary Data Sheet 5) was provided.

### The lymphocyte subgroup assay

The lymphocyte subgroups were measured by Flow cytometry (CytoFLEX flow cytometry system, Beckman coulter, Inc.) using commercially available kits (Beckman coulter, Inc.) according to the manufacture’s protocol. Briefly, the reagents of the BD six-color lymphocyte subgroup (FITC-CD3, PE-CD16/PE-CD56, PerCP-Cy5.5-CD45, PE-Cy7-CD4, APC-CD19, and APC-Cy7–CD8) were mixed with the whole blood and incubated at room temperature for 20 min, followed by adding 1 mL of a lysis solution with 30 min incubating. The proportion of CD3+, CD3+/CD4+, CD3+/CD8+, CD3−/CD19+, CD3−/CD56+/CD16+ cells in lymphocytes was analyzed with the software.

### Serum anti-SARS-CoV-2 antibodies assay

Total SARS-CoV-2 IgM or IgG in the serum was measured by chemiluminescence using commercially available kits (Shenzhen YHLO Biotech Co., Ltd.), which was coated with N and S proteins, in 1850 patients at different time points. In addition, 416 of these patients were tested for S-specific, RBD-specific, and N-specific IgM and IgG levels at different time points by chemiluminescence using commercially available kits (Nanjing RealMind Biotech Co., Ltd.). Briefly, the blood samples were centrifuged at room temperature, the supernatant was taken and incubated with antigen-coated magnetic beads. The antigen-antibody complex is then captured, incubated, and reacted with hydrogen peroxide in an excitatory buffer. Relative luminescence intensity was recorded in the ACL2800 chemrenaliluminescence system (Nanjing RealMind Biotech Co., Ltd.). The relative luminescence intensity was converted to AU/ML antibody levels. Relative antibody levels were presented as the measured chemiluminescence values divided by the constant derived from the linear correlation, which was signal-to-cutoff (S/CO). S/CO > 1 was defined as positive and S/CO ≤1 as negative (Nanjing RealMind Biotech Co., Ltd.). Similarly, S/CO > 10 was defined as positive and S/CO ≤10 as negative (Shenzhen YHLO Biotech Co., Ltd.). Also, we validated the performance of commercial kits for antibody detection. None of nine healthy controls, five patients infected with hepatitis B virus, or five patients with syphilis tested positive for S-IgM, S-IgG, RBD-IgM, RBD-IgG, N-IgM, or N-IgG. All of nine COVID-19 patients tested positive for S-IgM, S-IgG, RBD-IgM, RBD-IgG, N-IgM, or N-IgG (Supplementary Figure S1, Supplementary Data Sheet 6).

### Definition of physiological function abnormalities

Patients whose B-type natriuretic peptide (BNP) level was not within the normal range (0–100 pg/ml) (Supplementary Table S1) were defined as patients with abnormal cardiac function. Patients whose creatinine (CRE) level was not within the normal range (57–111 umol/L vs. 41–81 umol/L in males and females, respectively) were defined as patients with renal function abnormality. If any of the indicators, which were total bile acid (TBA, 0–10 umol/L), total bilirubin (TBIL, 0–26 umol vs. 0–21 umol in males and females, respectively), direct bilirubin (DBIL, 0–8 umol/L), indirect bilirubin (IBIL, 0–14 umol/L), glutamic-pyruvic transaminase (GPT, 9–60 IU/L), glutamic oxalacetic transaminase (GOT, 7–45 IU/L), and alkaline phosphatase (ALP, 45–125 IU/L vs. 35–135 IU/L in males and females, respectively) was not within the normal range, these patients were defined as patients with abnormal liver function.

### Statistical analysis

Statistical analysis was performed in R version 3.6.0. We used the Wilcoxon rank-sum test or Fisher’s exact test to compare the difference between groups where appropriate. Continuous and categorical variables were presented as median (IQR) and n (%), respectively.

Survival was estimated according to the Kaplan–Meier method by R package “survival”. The log-rank test was used to assess statistical significance.

To recognize the risk factors for death from COVID-19 patients, those variables associated with survival of COVID-19 patients (age, sex, pre-existing diseases, days from symptom onset to admission, and days from admission to discharge) were evaluated using univariable Cox regression models by “coxph” function in R package “survival”. *P*-value < 0.05 was considered statistically significant. Those variables with not significant P-value from the Wald test were removed one at a time, while the significant variables of univariable analysis were entered into the multivariable Cox proportional hazards model and analyzed by “coxph” function in R package “survival”. Also, P-value < 0.05 was considered statistically significant in multivariable analysis.

## Results

### Sex is an independent prognostic factor for COVID-19

**Fig. 1:**
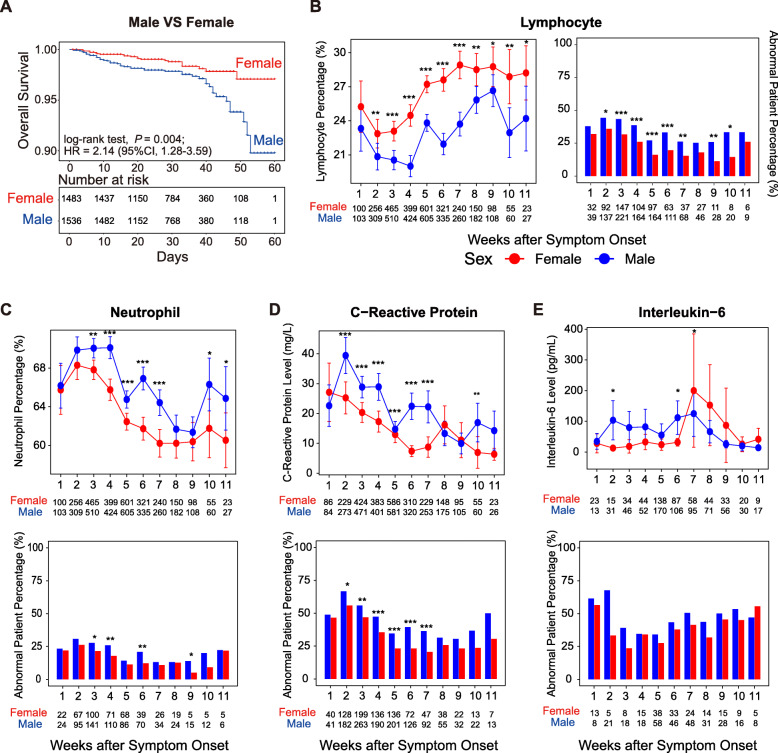
Survival analysis and comparison of prognostic related indicators between males and females from the first to 11th week after symptom onset. **A** Sex-based survival analysis of COVID-19 patients. **B**-**E** Differences of laboratory findings in male and female patients. Red represents female patients, and blue represents male patients. The x-axis displays weeks after symptom onset. The y-axis displays the level of indicators or percentage of patients with abnormal indicators. The line chart shows the mean and standard deviation of indicator values, and the significance is calculated by the Wilcoxon test. The Fisher test calculates the significance in the histogram. The number of patients per week after symptom onset is shown in the graph. *, *P* < 0.05; **, *P* < 0.01; ***, *P* < 0.001

To assess the impact of sex in COVID-19, we compared the clinical characteristics and outcomes between male and female patients (Table 1). There were more male patients with pre-existing chronic obstructive pulmonary disease compared to female patients (96 [6.2%] vs. 51 [3.4%], in males and females, respectively, *P* < 0.001). Besides, pre-existing chronic liver disease was more common in male patients (57 [3.7%] vs. 26 [1.7%], in males and females, respectively, *P* = 0.002). Although the hospitalization time had no significant difference between sex, the severity of COVID-19 patients was significantly associated with male sex (*P* = 0.002), with the percentage of critically ill patients higher in male patients than in female patients (6.2% vs. 3.5%, in males and females, respectively). During the hospitalization, 73 (4.69%) male and 41 (2.74%) female patients were eventually admitted to the ICU (*P* = 0.005). Notably, the mortality in male patients was more than 2-fold higher than that in female patients (3.0% vs. 1.40%, in males and females, respectively, *P* = 0.004). To deepen the understanding of the relationship between sex and prognosis, we performed survival analysis for 3057 COVID-19 patients (Fig. 1A). Results showed that male patients had a significant unfavorable prognosis (log-rank test, crude HR = 2.14, 95% CI: 1.28–3.59, *P* = 0.004). Moreover, by integrating age, sex, hospitalization time, and various pre-existing diseases to perform univariable and multivariable Cox Regression (Table 2), we found that male sex is an independent risk factor for death from COVID-19 patients (adjusted HR = 2.22, 95% CI: 1.31–3.74, *P* = 0.003). Older age and lower time from symptom onset to admission were also significant independent risk factors for death from COVID-19 patients.

**Table 1:** Comparison of Clinical Characteristics and outcomes between Males and Females

Characteristics	Total (***N*** = 3057)	Male (***N*** = 1558)	Female (***N*** = 1499)	***P***-value
**Age (yr.)– median **(IQR^*^)	60 (49–68)	60 (48–69)	60 (51–68)	0.2
**Pre-existing diseases – no. (%)**				
Hypertension	931 (30.5)	474 (30.4)	457 (30.5)	> 0.99
Diabetes	419 (13.7)	228 (14.6)	191 (12.7)	0.1
Cardiovascular disease	348 (11.4)	178 (11.4)	170 (11.3)	> 0.99
Cerebrovascular disease	124 (4.1)	68 (4.3)	56 (3.7)	0.4
Malignancy	80 (2.6)	42 (2.7)	38 (2.5)	0.8
Chronic obstructive pulmonary disease	147 (4.8)	96 (6.2)	51 (3.4)	***< 0.001***
Chronic renal disease	52 (1.7)	29 (1.9)	23 (1.5)	0.5
Chronic liver disease	83 (2.7)	57 (3.7)	26 (1.7)	***0.002***
Immunodeficiency	10 (0.3)	1 (0.06)	9 (0.6)	***0.02***
**Days from symptom onset to admission(d) – median (IQR)**	25 (14–35)	24 (14–35)	25 (15–35)	*0.1*
**Days from admission to** **discharge(d) – median (IQR)**	13 (8–19)	13 (8–19)	13 (8–19)	*0.9*
**Days from symptom onset to admission(d) – median (IQR)**	25 (14–35)	24 (14–35)	25 (15–35)	*0.2*
**Days from admission to** **discharge(d) – median (IQR)**	13 (8–19)	13 (8–19)	13 (8–19)	*0.8*
**Degree of severity – no. (%)**				***0.002***
Mild/Moderate	1455 (47.6)	739 (47.4)	716 (47.8)	
Severe	1417 (46.4)	700 (44.9)	717 (47.8)	
Critical	150 (4.9)	97 (6.2)	53 (3.5)	
**ICU admission – no. (%)**	114 (3.7)	73 (4.7)	41 (2.7)	***0.005***
**Clinical outcomes – no. (%)**				
Discharge from hospital	2940 (96.2)	1481 (95.1)	1459 (97.3)	***0.001***
Continual Cure	50 (1.6)	31 (1.9)	19 (1.3)	*0.1*
Death	67 (2.2)	46 (3.0)	21 (1.4)	***0.004***

IQR^*^ interquartile range

**Table 2:** Univariable and multivariable analyses of survival in COVID-19 patients

Variable	Univariable analysis			Multivariable analysis		
	HR^*^	95% CI^^^	***P*** value	aHR^#^	95% CI	***P*** value
**Age, years**	1.07	1.05–1.09	**< 0.001**	1.06	1.04–1.08	**< 0.001**
**Sex, male vs. female**	2.14	1.28–3.59	**0.004**	2.22	1.31–3.74	**0.003**
**Time from symptoms onset to admission, years**	0.93	0.91–0.95	**< 0.001**	0.93	0.91–0.95	**< 0.001**
**Days from admission to discharge, years**	0.99	0.97–1.02	0.6			
**Hypertension, yes vs no**	1.38	0.84–2.27	0.2			
**Diabetes, yes vs. no**	1.37	0.73–2.55	0.3			
**Cardiovascular disease, yes vs. no**	1.64	0.88–3.07	0.1			
**Cerebrovascular disease, yes vs. no**	1.41	0.51–3.89	0.5			
**Malignancy, yes vs. no**	1.76	0.55–5.61	0.3			
**Chronic obstructive pulmonary disease, yes vs.no**	1.35	0.49–3.7	0.6			
**Chronic renal disease, yes vs.no**	0.87	0.12–6.27	0.9			
**Chronic liver disease, yes vs. no**	1.15	0.28–4.68	0.9			

HR^*^ hazard ratio; *95% CI*^^^ 95% confidence interval; *aHR*^#^ adjusted hazard ratio

Next, by analyzing dynamic changes of laboratory indicators in COVID-19 patients after symptom onset of disease, we found that the lymphocyte percentage of female patients was relatively high in the first week after symptom onset and decreased in the second week after symptom onset, followed by a continuous increase to higher levels (Fig. 1B), whereas the lymphocyte percentage of male patients was lower than that of female patients during hospitalization. Also, we identified patients with abnormal lymphocyte percentages based on the normal range of laboratory findings (Supplementary Table S1). We found that the percentage of abnormal lymphocyte count in male patients was significantly higher than that of female patients in eight weeks out of eleven weeks (Fig. 1B). Male patients had a higher neutrophil percentage than female patients during hospitalization, and the percentage of male patients with abnormal neutrophil percentage was significantly higher than abnormal female patients in the third, fourth, sixth, and ninth week after symptom onset (Fig. 1C). The concentration of C-reactive protein (CRP) in male patients in the first week after symptom onset was similar to that in female patients. From the second week to the seventh week after symptom onset, the level of CRP in male patients was significantly higher as well as the percentage of male patients with abnormal CRP levels (Fig. 1D). Besides, the level of inflammatory cytokine interleukin-6 (IL-6) was higher in male patients between the second week to the sixth week after symptom onset (Fig. 1E). Similarly, we found that severe/critical patients had a lower percentage of lymphocytes, higher percentage of neutrophils, higher CRP level, and higher inflammatory cytokine level than mild/moderate patients. Compared with females of the same degree of disease severity, males had lower lymphocyte, higher neutrophil, and higher inflammatory cytokines levels (Supplementary Figure S2). Finally, we compared the number of patients with cardiac, renal, or hepatic function abnormality between male and female patients during hospitalization. Results showed that the percentage of renal (102/1588 [6.5%] vs. 63/1499 [4.2%], in males and females, respectively, *P* = 0.002) or hepatic abnormalities (650/1588 [40.9%] vs. 475/1499 [31.7%], in males and females, respectively, *P* = 0.003) was significantly higher in male patients than that in female patients (Supplementary Figure S3, Supplementary Table S2). These findings suggested that male patients had higher neutrophil, CRP, and inflammatory cytokine levels, and lower lymphocyte levels during hospitalization.

### Male patients have a lower percentage of CD4^+^ T and CD19^+^ B cells in whole blood during hospitalization

**Fig. 2:**
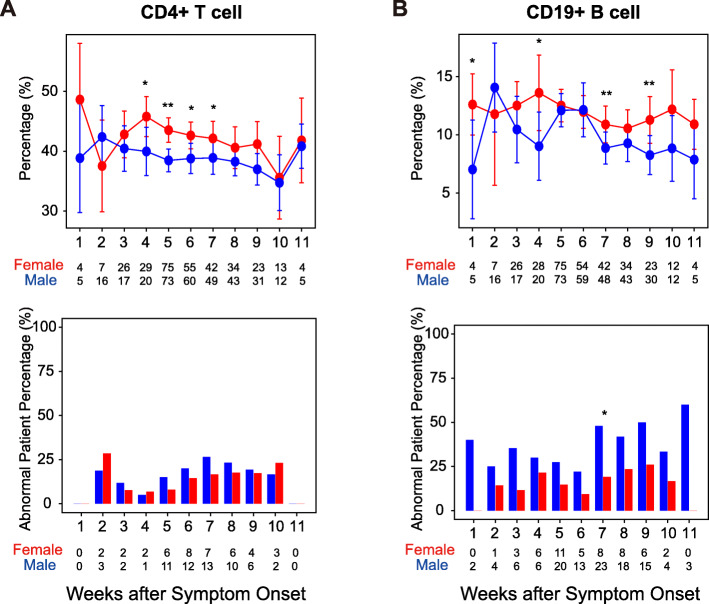
Comparison of lymphocyte subsets in peripheral blood between males and females from the first to 11th week after symptom onset. Red represents female patients, and blue represents male patients. The x-axis displays weeks after symptom onset. The y-axis displays the level of indicators or percentage of patients with abnormal indicators. The line chart shows the mean and standard deviation of indicator values. The line chart shows the mean and standard deviation of indicator values, and the significance is calculated by the Wilcoxon test. The Fisher test calculates the significance in the histogram. The number of patients per week after symptom onset is shown in the graph. *, *P* < 0.05; **, *P* < 0.01; ***, *P* < 0.001

To explore the immunological basis in male patients, we analyzed the lymphocyte subsets between sex, including whole blood percentages of CD4^+^ T cells, CD8^+^ T cells, CD19^+^ B cells, and CD16^+^CD56^+^ NK cells. Results showed that the percentage of CD4^+^ T cells in female patients was generally higher than in male patients during hospitalization. Specifically, it was significantly higher in the fourth, fifth, sixth, and seventh weeks after symptom onset in female patients (Fig. 2A). The percentage of CD8^+^ T cells was significantly higher in male patients than in female patients in the ninth week after symptom onset (Supplementary Figure S4A). Notably, the percentage of CD19^+^ B cells in female patients was higher than that in male patients during hospitalization. Specifically, it was significantly higher in the first, fourth, seventh, and ninth weeks after symptom onset. In addition, the percentage of female patients with abnormal CD19^+^ B cells percentage was significantly higher than male patients in the seventh week after symptom onset (Fig. 2B). During the course of COVID-19, the dynamic change pattern of CD56+ NK cells was similar between male and female patients (Supplementary Figure S4B). Generally, male patients had a lower percentage of CD4^+^ T and CD19^+^ B cells during hospitalization (Fig. 2), and the dynamic change patterns of CD4^+^ T cells or CD19^+^ B cells in male patients or female patients under different disease states were similar (Supplementary Figure S2). These results suggested that males had a decreased percentage of CD19^+^ B cells and CD4^+^ T cells as compared to females during the infection and recovery of COVID-19.

### The response of SARS-CoV-2 specific IgG is more rapid in female patients

**Fig. 3:**
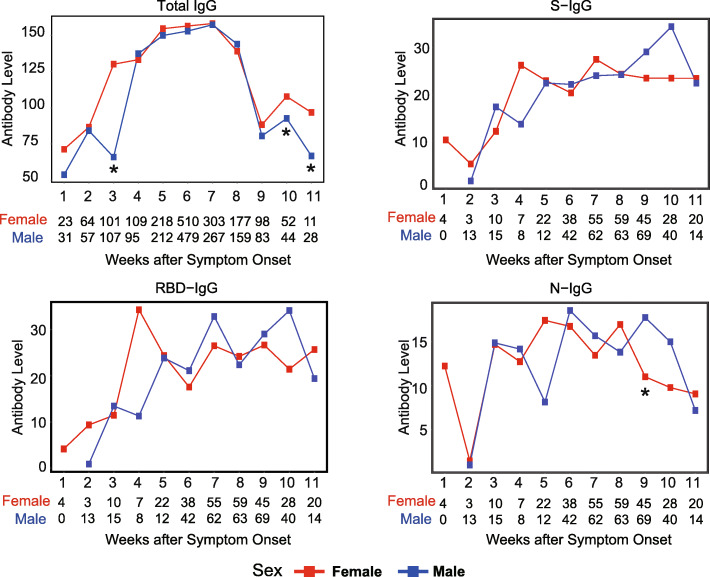
The dynamic changes of antibodies against SARS-CoV-2. The x-axis displays the weeks after symptom onset. The numbers below the figure represent the number of tests of females and males. The y-axis displays the level of IgG level. The red line based on the median is used to profile the females’ variation tendency, and the blue line based on the median is used to profile the males’ variation tendency. The significance is calculated by the Wilcoxon rank-sum test. The number of patient tests per week after symptom onset is shown in the graph. *, *P* < 0.05; **, *P* < 0.01; ***, *P* < 0.001

As the observed high percentage of CD4^+^ T cells and CD19^+^ B cells in females, we further compared the dynamic changes of the total, spike protein (S)-, receptor binding domain (RBD)-, and nucleoprotein (N)- specific IgM and IgG levels during SARS-CoV-2 infection and recovery between male and female patients (Fig. 3, Supplementary Figure S5). We observed similar dynamic trends of total IgM and IgG in male and female patients, except that the total IgG reached a relatively high level in the third week after symptom onset in female patients, while it took 4 weeks for the male patients to get the comparable antibody level. In addition, female patients had significantly higher total IgG levels than male patients in the third, tenth, and eleventh weeks after symptom onset. The N-specific IgG level showed a similar pattern between male and female patients. The RBD-specific IgG level sharply increased within the first 4 weeks after symptom onset in female patients. However, the RBD-specific IgG level increased more slowly in male patients, and it took at least 7 weeks for males to reach a comparable level of the fourth week after symptom onset in female patients. The RBD-specific IgG levels were 11.3 AU/ML and 34.3 AU/ML in the fourth week after symptom onset in males and females. Respectively. Moreover, the dynamic changes of the S-specific IgG level showed a similar trend with RBD-specific IgG. The level of S- specific IgM in female patients increased until the third week after symptom onset, and then gradually decreased, and was significantly lower than that in male patients at the seventh and eighth week after symptom onset. Similarly, the RBD- specific IgM level in female patients was lower compared with male patients at the seventh and eighth week after symptom onset.

## Discussion

Although previous epidemiological studies reported that the mortality of male COVID-19 patients was higher than female patients [12], the underlying cellular and molecular mechanisms have not been comprehensively analyzed due to the insufficient sample size of test data. In this study, using homogeneous data from a single-center, we described the clinical and laboratory characteristics of 3057 COVID-19 patients (1558 males and 1499 females) and performed a comparative analysis for clinical outcomes and immunological responses between males and females. Our results showed that the mortality, ICU admission rate, and percentage of critical cases were approximately 2-fold higher in males than that in females. Male sex is an independent prognostic risk factor for death from COVID-19. Previous studies reported that the ratio between neutrophil and lymphocyte percentage is an important index for the prognosis of COVID-19 [13, 14], and that patients with lower lymphocyte percentage and higher neutrophil percentage often have a poor outcome [15]. Our findings were consistent with this evidence, which implied that low lymphocyte and high neutrophil percentage are associated with poor prognosis of male patients. Moreover, the elevated level of cytokines (or even cytokine storm) could lead to acute pulmonary injury and acute respiratory distress syndrome (ARDS), related to ICU admission and death [16]. Meng Y et al. reported higher CRP levels in male patients [15] and Del Valle DM et al. reported higher IL-6 levels in male COVID-19 patients [17]. Our results are also consistent with their findings. The renal and hepatic abnormalities in COVID-19 patients were more common in male patients during the hospitalization, consistent with Meng Y et al.’s study which reported higher liver enzymes and lower kidney function in male COVID-19 patients [15]. In summary, male patients had higher neutrophil, CRP, and inflammatory cytokine levels, and lower lymphocyte levels, which were related to the poor prognosis and severe clinical symptoms of male COVID-19 patients. The humoral and cellular immune response played an important role in defending against SARS-CoV-2 infection [18] and helping in recovery from COVID-19. B cells play a pivotal role in humoral immunity by differentiating to plasma cells under the stimulation of foreign antigens, and plasma cells can synthesize and secrete specific antibodies against virus infection [19]. CD4+ T cells are indispensable in promoting the differentiation of B cells to plasma cells [20, 21]. Further immunological analysis revealed that male patients had a lower percentage of CD4^+^ T cells and CD19^+^ B cells than female patients during the infection and recovery of COVID-19, and the response of protective antibodies was slower in male patients than in female patients.

In this decade, it is increasingly acknowledged that males and females differ in their immunological responses to foreign and self-antigens, including both innate and adaptive immune responses [22]. Klein SL et al. reported higher antibody levels in convalescent male patients [23], suggesting that males might need more antibodies to recover from COVID-19. Our results indicated that although the S- and RBD- specific IgG levels continuously increased in male patients during the COVID-19 recovery, the response of these protective antibodies [24] was slower in male patients than in female patients. The indolent antibody responses in male patients may lead to their rapid disease progression. This result indicated the importance of early medical intervention for males with COVID-19. Furthermore, our previous studies reported that S and RBD specific IgG were protective antibodies against SARS-CoV-2, which was associated with virus shedding and improved severe clinical symptoms [15]. The level of protective antibody could be increased after convalescent plasma transfusion (CPT) [25]. Combined with this evidence, immunotherapy such as early plasma transfusion might enhance the immunity of male patients by improving the original antibody level. Also, research found that severe or critical patients had higher antibody levels during hospitalization than mild or moderate patients [26]. Therefore, close monitoring of various indicators is necessary, especially for severe male patients.

## Conclusions

In this study, by analyzing the sex-based clinical and immunological differences in a large COVID-19 cohort, we found that male patients had an unfavorable prognosis, higher inflammation, lower percentage of lymphocytes, and indolent antibody responses during SARS-CoV-2 infection and recovery from COVID-19. Meanwhile, male patients need early intervention and close monitoring. Our results provided important information for the epidemiology and precise medical intervention for COVID-19, and shed new light on the development of immunological therapy for male patients.

## Supplementary Information

**Additional file 1: Supplementary Figure S1**. Validation of the performance of commercial kits.

**Additional file 2: Supplementary Figure S2**. Differences of laboratory findings in male and female patients based on the degree of severity.

**Additional file 3: Supplementary Figure S3**. The comparison of male and female patients with cardiac, hepatic, and renal abnormality.

**Additional file 4: Supplementary Figure S4**. Comparison of lymphocyte subsets in peripheral blood between males and females from the first to 11th week after symptom onset.

**Additional file 5: Supplementary Figure S5**. The dynamic changes of IgM levels.

**Additional file 6: Supplementary Table S1**. The normal range of laboratory indicators.

**Additional file 7: Supplementary Table S2**. Abnormal indicators between male and female patients.

**Additional file 8: Supplementary Data.**

## Data Availability

The datasets used and/or analysed during the current study are available from the corresponding author on reasonable request.

## Abbreviations

ALP: Alkaline phosphatase
ARDS: Acute respiratory distress syndrome
BNP: B-type natriuretic peptide
CI: Confidence interval
COVID-19: Coronavirus disease 2019
CPT: Convalescent plasma transfusion
CRE: Creatinine
CRP: C-reactive protein
DBIL: Direct bilirubin
GGT: Glutamyl transpeptidase
GOT: Glutamic oxalacetic transaminase
GPT: Glutamic-pyruvic transaminase
HR: Hazard ratio
IL-6: Interleukin-6
N: Nucleoprotein
RBD: Receptor binding domain
S: Spike protein
SARS-CoV-2: Severe acute respiratory syndrome coronavirus 2
TBA: Total bile acid
TBIL: Total bilirubin
