# Supplementary material for: Sex-based clinical and immunological differences in COVID-19
Source: BMC Infect Dis. 2021 Jul 5;21:647. doi: 10.1186/s12879-021-06313-2 (PMC8256650; doi:10.1186/s12879-021-06313-2)
Supplement: Supplementary file 1 — Additional file 1: Supplementary Figure S1. Validation of the performance of commercial kits. [file 12879_2021_6313_MOESM1_ESM.pdf]

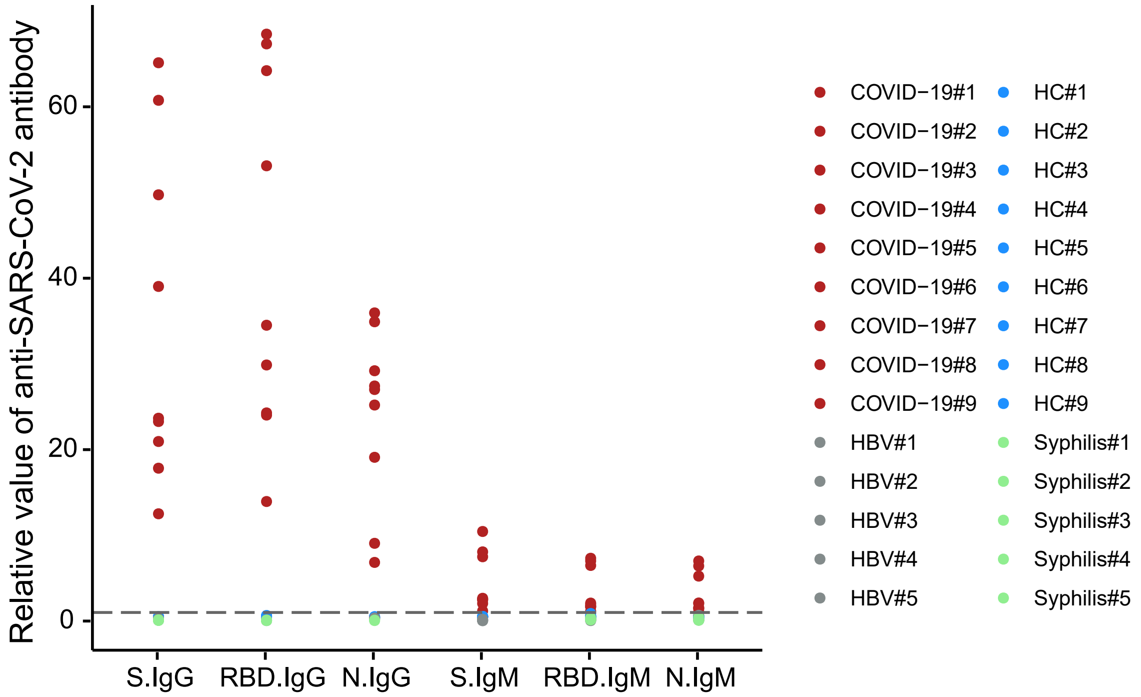

Supplementary Figure S1. Validation of the performance of commercial kits. The x-axis represents the antibody category, and the y-axis represents relative value of anti-SARS-CoV-2 antibody.
