# Supplementary material for: Sex-based clinical and immunological differences in COVID-19
Source: BMC Infect Dis. 2021 Jul 5;21:647. doi: 10.1186/s12879-021-06313-2 (PMC8256650; doi:10.1186/s12879-021-06313-2)
Supplement: Supplementary file 2 — Additional file 2: Supplementary Figure S2. Differences of laboratory findings in male and female patients based on the degree of severity. [file 12879_2021_6313_MOESM2_ESM.pdf]

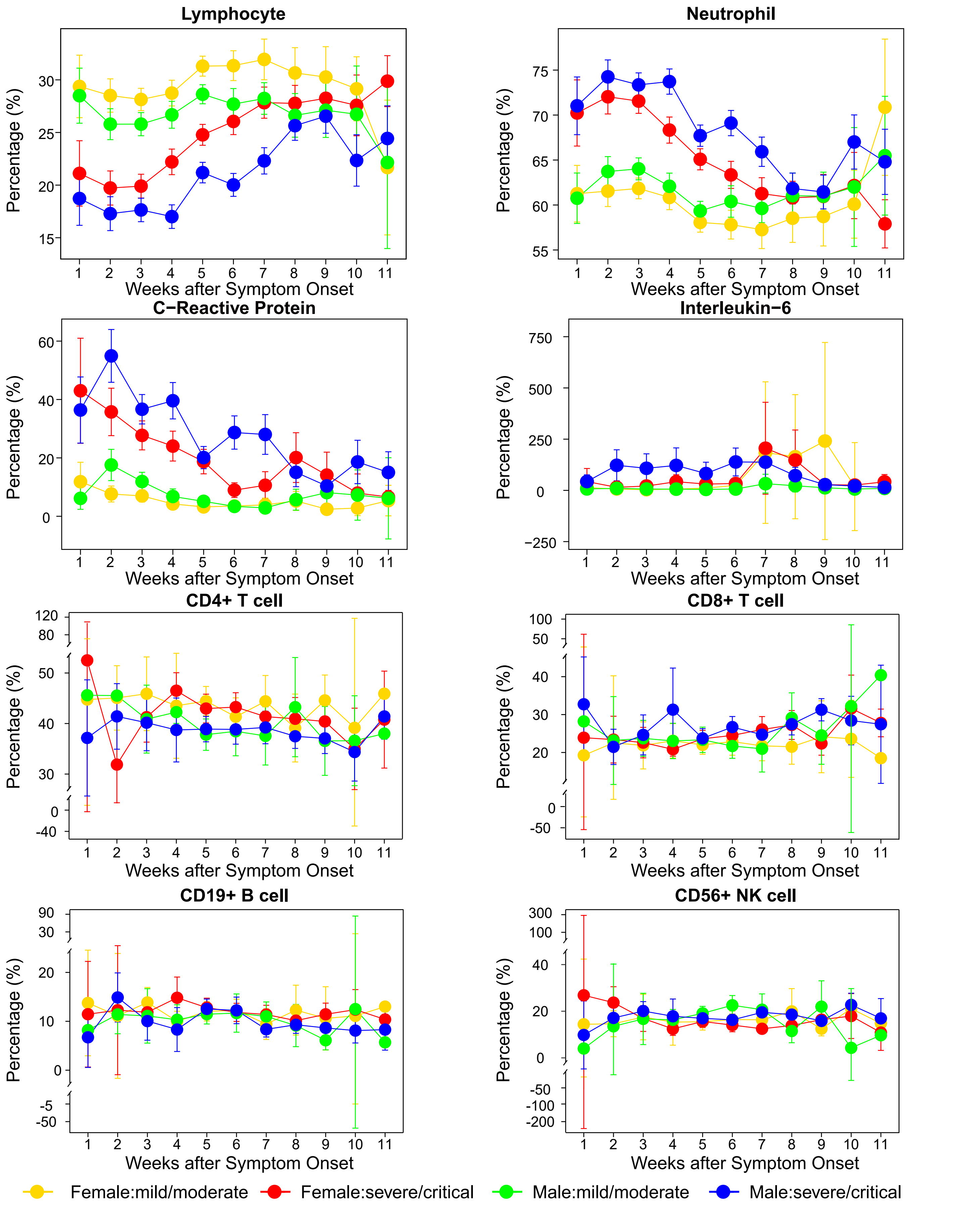

Supplementary Figure S2. Differences of laboratory findings in male and female patients based on the degree of severity. The x-axis displays weeks after onset. The y-axis displays the level of indicators. The line chart shows the mean and standard deviation of indicator values.
