# Supplementary material for: Sex-based clinical and immunological differences in COVID-19
Source: BMC Infect Dis. 2021 Jul 5;21:647. doi: 10.1186/s12879-021-06313-2 (PMC8256650; doi:10.1186/s12879-021-06313-2)
Supplement: Supplementary file 3 — Additional file 3: Supplementary Figure S3. The comparison of male and female patients with cardiac, hepatic, and renal abnormality. [file 12879_2021_6313_MOESM3_ESM.pdf]

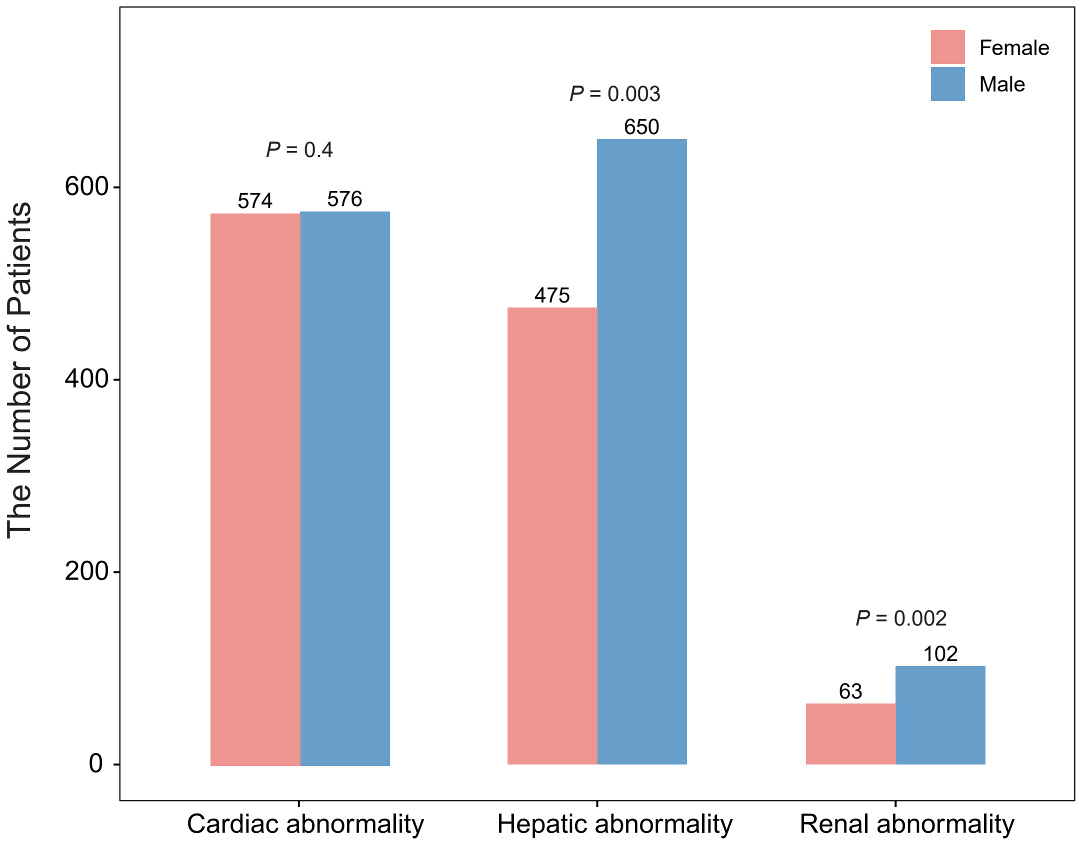

Supplementary Figure S3. The comparison of male and female patients with cardiac, hepatic, and renal abnormality.
