# Supplementary material for: Sex-based clinical and immunological differences in COVID-19
Source: BMC Infect Dis. 2021 Jul 5;21:647. doi: 10.1186/s12879-021-06313-2 (PMC8256650; doi:10.1186/s12879-021-06313-2)
Supplement: Supplementary file 4 — Additional file 4: Supplementary Figure S4. Comparison of lymphocyte subsets in peripheral blood between males and females from the first to 11th week after symptom onset. [file 12879_2021_6313_MOESM4_ESM.pdf]

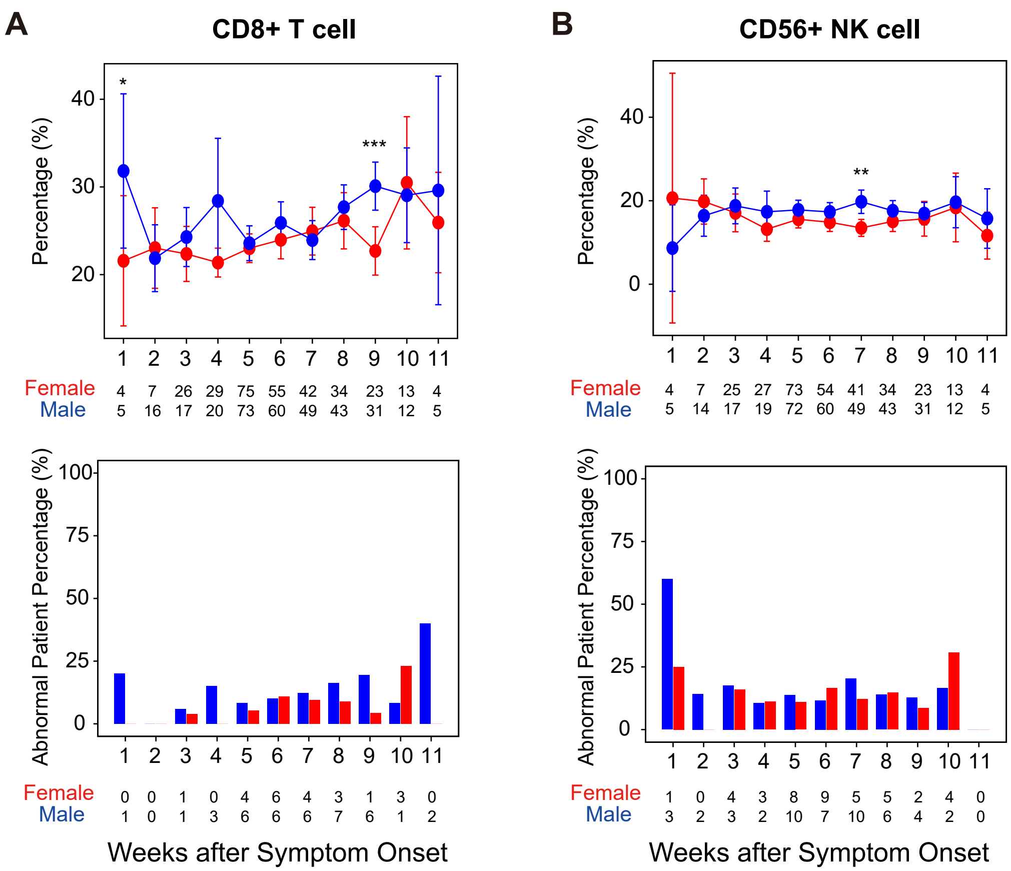

Supplementary Figure S4. Comparison of lymphocyte subsets in peripheral blood between males and females from the first to 11th week after symptom onset. Red represents female patients, and blue represents male patients. The x-axis displays weeks after onset. The y-axis displays the level of indicators or percentage of patients with abnormal indicators. The line chart shows the mean and standard deviation of indicator values. The line chart shows the mean and standard deviation of indicator values, and the significance is calculated by the Wilcoxon test. The significance in the histogram is calculated by the Fisher test. The number of patients per week after onset is shown in the graph. *, $P < 0.05$; **, $P < 0.01$; ***, $P < 0.001$.
