# Supplementary material for: Sex-based clinical and immunological differences in COVID-19
Source: BMC Infect Dis. 2021 Jul 5;21:647. doi: 10.1186/s12879-021-06313-2 (PMC8256650; doi:10.1186/s12879-021-06313-2)
Supplement: Supplementary file 5 — Additional file 5: Supplementary Figure S5. The dynamic changes of IgM levels. [file 12879_2021_6313_MOESM5_ESM.pdf]

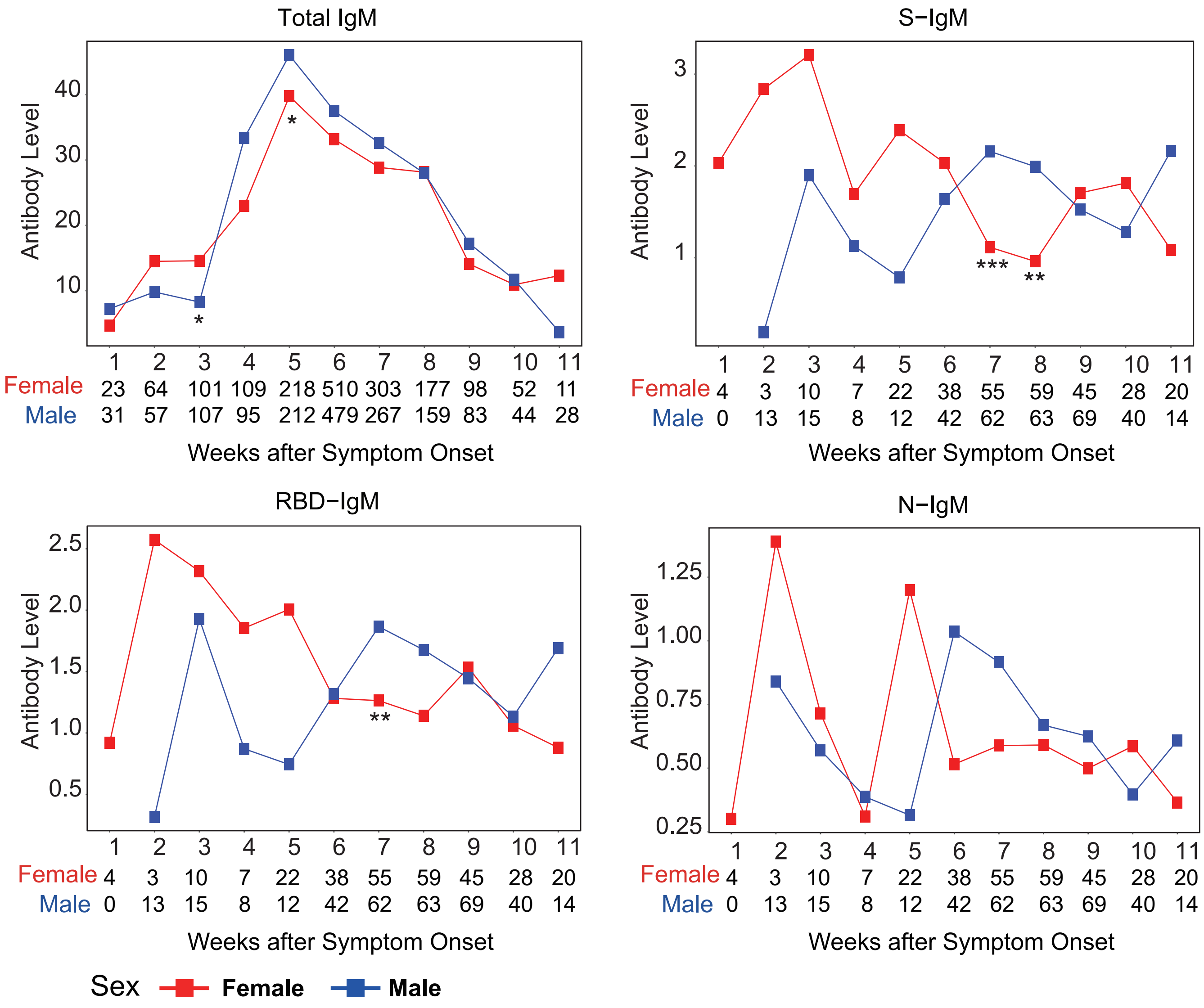

Supplementary Figure S5. The dynamic changes of IgM levels. The x-axis displays the weeks after admission. F indicates the number of tests of females, and M indicates the number of tests of males. The y-axis displays the level of IgM level. Red line based on median is used to profile the variation tendency of the females, and blue line based on median is used to profile the variation tendency of the males, The number of patient tests per week after symptom onset is shown in the graph. *, P < 0.05; **, P < 0.01; ***, P < 0.001.
