# Supplementary material for: Sex-based clinical and immunological differences in COVID-19
Source: BMC Infect Dis. 2021 Jul 5;21:647. doi: 10.1186/s12879-021-06313-2 (PMC8256650; doi:10.1186/s12879-021-06313-2)
Supplement: Supplementary file 6 — Additional file 6: Supplementary Table S1. The normal range of laboratory indicators. [file 12879_2021_6313_MOESM6_ESM.docx]

**Supplementary Table S1.** The normal range of laboratory indicators

| **Clinical indicators** | **Normal Range** | **Unit** |
| --- | --- | --- |

| Total bilirubin | 0-21 for female; 0-26 for male | umol/L |
| --- | --- | --- |
| Total bile acid | 0-10 | umol/L |
| Indirect bilirubin | 0-14 | umol/L |
| Glutamic-pyruvic transaminase | 9-60 | IU/L |
| Glutamic oxalacetic transaminase | 7-45 | IU/L |
| Creatinine | 41-81 for female; 57-111 for male | umol/L |
| Direct bilirubin | 0-8 | umol/L |
| B-type natriuretic peptide | 0-100 | pg/ml |
| Alkaline phosphatase | 35-135 for female; 45-125 for male | IU/L |
| C-reactive protein | 0-4 | mg/L |
| Interleukin 6 | <7 | pg/mL |
| Lymphocyte percent | 20-50 for female; 40-60 for male | % |
| Neutrophilic granulocyte percent | 40-75 for female; 31-40 for male | % |
| CD3+CD4+ T cell | 31-61 for female; 29-57 for male | % |
| CD3+CD8+ T cell | 11-38 | % |
| CD3-CD19+ B cell | 6.4-23 | % |
| CD3-(CD16+/CD56+) cell | 5.6-31 | % |
