# Supplementary material for: Sex-based clinical and immunological differences in COVID-19
Source: BMC Infect Dis. 2021 Jul 5;21:647. doi: 10.1186/s12879-021-06313-2 (PMC8256650; doi:10.1186/s12879-021-06313-2)
Supplement: Supplementary file 7 — Additional file 7: Supplementary Table S2. Abnormal indicators between male and female patients. [file 12879_2021_6313_MOESM7_ESM.docx]

**Supplementary Table S2.** Abnormal indicators between male and female patients

| Laboratory Findings-median (IQR) | Male  (N=1447) | Female  (N=1497) | P-value |
| --- | --- | --- | --- |
| B-type natriuretic peptide (BNP) pg/ml | 14.0 (0.0-91.5) | 13.0 (0.0-57.7) | 0.045 |
| Creatinine (CRE) umol/L | 72.7 (63.5-85.2) | 56.0 (49.9-64.6) | ***<0.001*** |
| Glutamic oxalacetic transaminase (GOT) IU/L | 30.6 (19.5-50.8) | 20.0 (13.2-31.8) | ***<0.001*** |
| Glutamic-pyruvic transaminase (GPT)  IU/L | 22.0 (17.2-32.0) | 19.4 (15.4-26.3) | ***<0.001*** |
| Glutamyl transpeptidase (GGT) IU/L | 38.5 (25.9-66.0) | 27.9 (18.4-45.9) | ***<0.001*** |
| Alkaline phosphatase (ALP) IU/L | 74.2 (61.9-91.6) | 71.4 (58.5-86.6) | ***<0.001*** |
